# Prolactin and Psychopathology in Schizophrenia: A Literature Review and Reappraisal

**DOI:** 10.1155/2014/175360

**Published:** 2014-03-27

**Authors:** Ravi Philip Rajkumar

**Affiliations:** Department of Psychiatry, Jawaharlal Institute of Postgraduate Medical Education and Research (JIPMER), Dhanvantari Nagar, 605 006 Pondicherry, India

## Abstract

Secretion of the anterior pituitary hormone prolactin can be significantly increased by antipsychotic drugs, leading to a range of adverse effects in patients with schizophrenia. However, there is evidence from a variety of studies that prolactin may also be related to symptom profile and treatment response in these patients, and recent work has identified variations in prolactin secretion even in drug-free patients. In this paper, a selective review of all relevant studies pertaining to prolactin and schizophrenia, including challenge and provocation studies, is presented. The implications of this work are discussed critically. A tentative model, which synthesizes these findings and argues for a significant role for prolactin in the development of schizophrenia, is outlined.

## 1. Introduction

Prolactin is a polypeptide hormone secreted by the anterior pituitary gland. Prolactin has multiple functions, including lactation and maternal-infant bonding, in mammals. Recent work has found it to be relevant to parental and sexual behaviour in humans [1, 2] as well. Various factors, including gender, sexual activity, childbirth, stress, smoking, and drugs, can affect the release of prolactin [3, 4].

The production of prolactin is inhibited by dopamine release in the hypothalamo-pituitary circuit and can be increased by blocking type 2 (D_2_) dopamine receptors. Most available antipsychotic drugs can therefore cause elevations in prolactin secretion. This increase is associated with a variety of adverse effects: lack of libido and erectile dysfunction in men [5], amenorrhoea and galactorrhoea in women [6], acceleration of osteoporosis in women [7], weight gain [8], and—potentially—an increased risk of cancer, particularly breast cancer in women [9]. The association of prolactin with male sexual dysfunction is complex and has been challenged by some authors [10] but is supported by research showing that antipsychotics which cause greater elevations in prolactin also have more marked sexual adverse effects [11]. Besides this, a variety of studies over the past four decades have examined other facets of the relationship between prolactin and schizophrenia and call for a reappraisal of this relationship. In this paper, the author highlights the important findings pertaining to prolactin and schizophrenia, excluding the literature on drug-induced hyperprolactinemia which has been extensively reviewed elsewhere. The author also proposes a model that may explain these findings. In the discussion that follows, the term “prolactin levels” refers to plasma prolactin, unless otherwise specified.

## 2. Studies of Prolactin Levels in Drug-Naïve Patients

As antipsychotic treatment generally causes hyperprolactinemia, measuring prolactin levels in drug-naïve patients is a useful method of assessing their relationship with schizophrenia itself. These studies are summarized in Table 1.

As seen from Table 1, the results of these studies are equivocal and are confounded by various methodological issues, including sampling time, gender, the use of medications, and the form of prolactin being assayed (total plasma, bioactive, or CSF). The two most recent studies to be published both found elevated plasma prolactin in drug-naïve patients with first-episode psychosis, while older studies found lower or equivalent levels.

## 3. Studies Reporting Relationships between Prolactin and Symptom Profile

The next question to be considered is whether elevated prolactin is related to specific symptoms or dimensions of schizophrenia, which is a multidimensional entity. Results of these studies are described in Table 2.

Examining the above studies yields a clearer, though not unequivocal, picture: there is a fairly consistent and negative association between plasma prolactin and surrogate measures of positive symptoms—such as specific delusions or the paranoid subtype of schizophrenia. Segal et al. [12] have interpreted these results in terms of increased dopaminergic tone in patients with paranoid symptoms, which is consistent with the dopamine hypothesis of schizophrenia. However, our interpretation remains confounded by the presence of studies with negative findings and by the variations in methodology adopted by different authors—particularly in terms of patient gender and the measures used to assess symptom dimensions.

## 4. Challenge and Provocative Test-Based Studies

Given the inconsistent results of studies measuring basal plasma prolactin, another fruitful line of enquiry would be assessing the prolactin response to various external stimuli in patients with schizophrenia, as these would give us a clearer notion of the physiological processes implicated in the altered levels. A variety of drugs—hormones, adrenergic agonists, serotonergic agonists, and dopamine antagonists—have been used to study prolactin release in patients with schizophrenia. The results of these studies are outlined in Table 3.

Some consistent findings emerge across these studies. A blunted prolactin response to pharmacological challenge in schizophrenia is documented across various classes of agents—adrenergic [13], serotonergic [14, 15], and dopaminergic [16–20]. However, these results are confounded by variables such as the dose of the pharmacological challenge, the effects of treatment, age and gender, and the duration of psychosis, and negative or inverse results have also been reported. With reference to drugs that inhibit prolactin release, results are more consistent, with many studies reporting a reduced suppression in patients [21–23]. Only one study has examined the effects of stress in this patient population and found a significantly greater increase in stress-stimulated prolactin in schizophrenia [24].

Relations with specific symptom dimensions have not been found consistently across studies. Positive correlations have been reported between the prolactin response to a serotonergic challenge and negative symptoms [25] as well as affective symptoms [26] and between prolactin release after TRH administration and thought disorder [27]. Negative correlations have been reported between prolactin response and inattention [28] as well as the severity of delusions [29]. Blunted prolactin responses have been associated with a “Kraepelinian” diagnosis of schizophrenia [17] and with schizophreniform disorder [20] in particular. A greater prolactin response to fenfluramine was associated with treatment resistance in one study [30]. Incidentally, one study found an association between a lower basal prolactin and positive symptoms [31], which is consistent with the results of Segal's studies [12, 32].

The interpretation of these findings is complicated by the wide range of potential mechanisms involved. The blunting of the prolactin response to dopamine antagonists [16–18] may indicate dopamine receptor supersensitivity, while the worsening of positive symptoms [15] associated with a blunted response to serotonergic challenge may implicate reduced serotonin receptor sensitivity in these symptoms. In contrast, the exaggerated prolactin response to serotonin in resistant schizophrenia is indicative of serotonergic hyperfunction; this is consistent with the fact that clozapine, an antagonist at multiple serotonin receptors, is the treatment of choice in this population.

## 5. Studies Reporting Relationships between Prolactin and Treatment Response

As prolactin elevation is related to D_2_ receptor blockade, prolactin may be a useful surrogate marker of the blockade achieved and thereby—in an indirect manner—of the efficacy of antipsychotic medications. Studies that have focused on this association are summarized in Table 4.

The results of the above studies suggest that there is a clear relationship between* changes* in prolactin level and the response to certain antipsychotics, with the best evidence pertaining to risperidone. The equivocal results obtained with other drugs may be related to the observation that prolactin has little predictive value beyond a certain “plateau” level [33] and may also reflect variables such as the effects of gender [34], the use of drugs with low dopamine antagonist activity [35], short treatment durations [36], small sample sizes [36–40], and the use of high doses of depot antipsychotics [38]. The suggestions that challenge tests can be used to assess the serotonergic effects of clozapine [41] and that plasma prolactin can be used to estimate therapeutic doses of dopamine antagonists [33] are both intriguing but require replication. Of note, some of the most relevant positive results have been obtained in male patients [33, 42, 43], suggesting that the utility of prolactin as a biomarker of drug response may be gender-specific.

## 6. Studies Reporting Relationships between Prolactin and Tardive Dyskinesia

As tardive dyskinesia (TD) is linked to dopamine receptor supersensitivity [44], alterations in prolactin levels would be expected in these patients. This relationship has been examined by a small number of studies, listed in Table 5.

Because of the small sample sizes involved, the results of these studies must be interpreted with caution. They may indicate dopamine receptor supersensitivity [45] or GABA receptor dysfunction [46], but variables such as gender, duration of illness, the nature of medications used, and the study design (basal prolactin versus challenge tests) must also be taken into account. With the available evidence, the association between prolactin and TD seems slight at best.

## 7. Studies Linking Prolactin with Circadian Rhythms

Alterations in genes regulating circadian rhythm have been linked to both schizophrenia and the affective disorders [47]. Like other hormones, prolactin shows circadian variations in its rhythm and may be used to track circadian rhythm changes in schizophrenia. These studies are summarized in Table 6.

These studies suggest that there are clear alterations in the circadian rhythm of prolactin in schizophrenia, particularly with regard to sleep [48, 49]. The possibility that schizophrenia has a unique circadian profile of prolactin and sleep changes, distinct from those seen in depression [49], must be seriously considered but requires further replication in larger samples using a consistent study protocol.

## 8. Prolactin and Suicide

Suicide attempts and completed suicide are not uncommon in schizophrenia [50]. A link between the prolactin response to a serotonergic challenge and affective symptoms, such as depression and guilt, has been reported in one study [26]; given this, it is logical to examine the association between prolactin levels and suicide. In a study examining 439 patients, 118 of whom were diagnosed as having psychosis, Pompili et al. [51] found slightly reduced prolactin levels in those patients who had attempted suicide.

## 9. Genetic Studies of Prolactin in Schizophrenia

Two recent studies have examined genetic variants in prolactin in patients with schizophrenia. Rybakowski et al. [52] examined the frequency of the −1149 G/T functional polymorphism of the prolactin gene in 403 patients, compared to 653 healthy controls. They found that the G allele was significantly more common in patients, particularly in males, and pointed out that this variation was similar to that reported in autoimmune diseases. Souza et al. [53] examined the association between the prolactin and prolactin receptor (PRLR) genes and both tardive dyskinesia and treatment response but failed to find a significant association in either case.

## 10. Summary and Hypothesis

The literature reviewed above, though complex, suggests fruitful lines for further enquiry. First, the association between raised prolactin and acute episodes of schizophrenia needs to be replicated in larger samples. Riecher-Rössler et al. [54] provide an intriguing explanation for this finding. They suggest that stress leads to an increased level of prolactin, which triggers dopamine release by a feedback mechanism; this increase in dopamine transmission may mediate the link between stress and psychosis. There are several lacunae in this hypothesis: (1) neither of the two studies cited above has measured stress or recent life events; (2) while raised prolactin may increase dopamine activity in the tuberoinfundibular pathway, it is not known if it can produce similar changes in the mesolimbic pathway; (3) this proposal fails to explain why several studies found lower or normal prolactin levels. Despite this, the hypothesis merits further testing, as well as extension to conditions such as acute and transient psychosis, where the role of stress is much clearer. The finding that patients with schizophrenia have an exaggerated prolactin response to stress may be relevant here.

Second, the association of lower prolactin levels with positive symptoms and “paranoid” symptoms in particular are in line with the revised dopamine hypothesis of schizophrenia. This association can certainly be interpreted as suggesting increased dopamine transmission in this subgroup, with the same caveat as above: we do not know how well prolactin correlates with dopamine activity in the mesolimbic pathway. If this finding can be replicated, it provides some support for the notion of subtypes within the broad category of schizophrenia. Given that the most recent edition of the DSM has consciously omitted the traditional subtypes [55], a biomarker for a paranoid subtype could lead to a reversion of this change.

Third, provocation studies provide further support for the idea of blunted or lowered prolactin secretion in patients with “positive” schizophrenia. Whether this relationship holds good for other conditions in which “positive” symptoms are prominent—such as psychotic mood disorders or acute and transient psychosis—needs to be decided by further research. Though alterations in dopamine, serotonin, and GABA activity are all hinted at by individual provocation studies, the true nature of these abnormalities is likely to be slight and subtle, and their significance is unclear. A similar conclusion would apply to the results of studies of circadian rhythm.

Fourth, the association between prolactin and treatment response to antipsychotics, as well as its relationship to tardive dyskinesia, is inconsistent and, in the latter case, unlikely to be of much clinical significance. Given the numerous confounders that can affect prolactin levels, the inconsistency of positive findings and the negative results of recent genetic research, it is unlikely that a consistent association can be made. Changes in prolactin level may be a more sensitive indicator of response in some cases, though these probably reach a plateau at higher doses and may depend crucially on the drug being studied.

Finally, the association of prolactin gene variants with schizophrenia—though preliminary—suggests that there is more to the schizophrenia-prolactin association than simple dopamine-based models. Though Rybakowski et al. [52] invoked the possibility of an autoimmune mechanism, another possibility exists: genetic variations in prolactin are a genuine risk factor for psychosis. As authors writing in other contexts have pointed out, prolactin has a bewildering array of effects on the behaviour of mammals, but its exact behavioural effects in humans are unknown [2]. Given its relationship with parental bonding, an effect of prolactin on social cognition and behavior—both of which are significantly impaired in schizophrenia—is plausible. A tentative model could run as follows. First, genetic variations in prolactin lead to an exaggerated prolactin response to stress, which may further be modulated by childhood adversity or possibly by substance abuse [56, 57]. This is supported by the results of recent research which has found elevated prolactin levels not only in first-episode psychosis, but in over 25% of patients with “at-risk” or prodromal psychosis [58]; the authors of this paper have also suggested, in line with the above model, that this could reflect a stress response. Second, when exposed to further stressors, increases in prolactin levels may indirectly—as in Riecher-Rossler's proposal of a feedback increase in dopamine, which may also be exaggerated—lead to some of the symptoms of psychosis. Third, as a result of subsequent changes, which again may include increased dopamine transmission, prolactin levels decline, and lower prolactin levels may correlate with the development of positive symptoms, such as delusions. This is consistent with both the possibility that prolactin influences social behaviour, and the finding that some forms of stress, such as childhood neglect [59] and social defeat [60], can sensitize the mesolimbic dopamine pathway. Fourth, a moderate increase in prolactin, brought about by antipsychotics, may play a role in alleviating the positive symptoms of schizophrenia. A role for higher cortical centres in modulating prolactin release, which would be consistent with the above, is suggested by the results of studies of lobotomized patients. This hypothesis complements the dopamine model of schizophrenia and is consistent with most of the evidence reviewed above. Further research into the genetic links between prolactin and schizophrenia, the exact relationship between prolactin and central dopamine, the significance of circadian changes in prolactin, and the effects of prolactin on human social behaviour will provide a true test of this proposal.

## 11. Conclusion

A review of the literature on prolactin and schizophrenia suggests that the relationship between them is complex and not confined to the adverse effects of antipsychotics. Though the above interpretations must be regarded as imperfect and tentative, they do call for a reappraisal of the role of prolactin in the various stages of schizophrenia, particularly with regard to its onset and to the development of positive symptoms. Research in this area may lead to an improved understanding of schizophrenia, as well as a better delineation of the effects of prolactin on social behaviour and cognition in humans.

## Figures and Tables

**Table 1:** Studies measuring prolactin levels in schizophrenia.

Study and authors	Patient sample	Results
Garcia-Rizo et al., 2012 [61]	Newly diagnosed patients with first-episode, nonaffective psychosis; comparison group of matched healthy controls	Significant elevation in prolactin even after controlling for confounders; more marked in women

Riecher-Rössler et al., 2013 [54]	74 antipsychotic-naïve patients with first-episode psychosis	39% of the study subjects had hyperprolactinemia

Warner et al., 2001 [62]	7 drug-free male patients with schizophrenia; 7 male controls	Lower levels of bioactive prolactin in the patient group

Rao et al., 1984 [63]	10 acutely ill patients with schizophrenia; 10 healthy controls	Lower levels of prolactin in patients; the authors also found lower levels of TSH and L-thyroxine

Kuruvilla et al., 1993 [64]	116 patients with schizophrenia; 120 healthy controls	No difference in prolactin levels between the two groups

Muck-Seler et al., 2004 [65]	20 women with schizophrenia drug-free for 7 days, compared with 25 depressed and 25 normal women	No difference in prolactin levels across the three groups

Rimon et al., 1981 [66] *(CSF prolactin) *	28 lobotomized and 28 nonlobotomized patients with “chronic schizophrenia”	Higher CSF prolactin levels in women and in nonlobotomized patients

Hyyppä et al., 1980 [67] *(CSF prolactin) *	Lobotomized and nonlobotomized patients with “chronic schizophrenia”	Higher CSF prolactin levels in nonlobotomized patients

**Table 2:** Studies examining the relationship between symptom profile and prolactin in schizophrenia.

Study and authors	Patient sample	Results
Segal et al., 2007 [12]	57 unmedicated male patients with schizophrenia (off medication for at least 3 months); 32 controls	Significantly lower prolactin levels in patients with the paranoid subtype of schizophrenia

Segal et al., 2004 [32]	48 first-episode and 38 recurrent patients with schizophrenia	Significantly lower prolactin levels in paranoid schizophrenia compared to schizoaffective and disorganized subtypes; no effect of illness duration on levels

Segal et al., 2007 [68]	45 male patients with schizophrenia, receiving risperidone	Significantly greater elevation of prolactin in patients with the paranoid subtype of schizophrenia

Otani et al., 1996 [69]	56 unmedicated patients with schizophrenia (28 male, 28 female)	Weak negative correlation between prolactin and hostility scores; no relation with other measures of psychopathology on the Brief Psychiatric Rating Scale

Newcomer et al., 1992 [70]	24 patients with schizophrenia on maintenance treatment with haloperidol	Significant positive correlation between prolactin levels and negative symptoms

Akhondzadeh et al., 2006 [71]	54 male patients with schizophrenia on maintenance treatment with haloperidol or risperidone; 25 healthy male controls	Significant positive correlation between prolactin levels and negative symptoms

Rinieris et al., 1985 [72]	Male patients with paranoid schizophrenia with or without “homosexual delusions”; healthy heterosexual controls	Lower prolactin levels associated with delusions having a homosexual content

Johnstone et al., 1977 [73]	16 unmedicated male patients with “chronic schizophrenia”	Serum prolactin was negatively correlated with both speech incoherence and total positive symptoms

Kleinman et al., 1982 [74]	17 drug-free patients with “chronic schizophrenia”	Inverse relationship between prolactin and total psychotic symptoms but only in those patients who did not show ventricular enlargement on computed tomography

Csernansky et al., 1986 [75]	33 male patients with schizophrenia on treatment; 8 off treatment; 18 normal male controls	“Prolactin index” (plasma prolactin divided by plasma neuroleptic activity) was negatively correlated with paranoid symptoms in younger patients receiving treatment

Prasad, 1986 [76]	13 patients off medication for 12 months: 4 with positive symptoms, 9 with negative symptoms	Higher prolactin levels in the group with positive symptoms

Luchins et al., 1984 [77]	23 patients with schizophrenia	No relationship between prolactin levels and psychopathology

**Table 3:** Results of studies examining prolactin response to pharmacological challenges in schizophrenia.

Study and authors	Agent used	Patient sample	Results
Spoov et al., 2010 [28]	Thyrotropin-releasing hormone (TRH), 12.5 mcg i.v.	19 drug-naïve patients with schizophrenia	Prolactin response negatively correlated with poverty of speech and inattention

Spoov et al., 1991 [27]	TRH, 12.5 mcg i.v.	20 patients with nonaffective psychosis	Prolactin response to TRH positively correlated with “nonparanoid” symptoms, such as thought disorder

Cabranes et al., 1982 [78]	TRH	Patients with acute and chronic schizophrenia treated with chlorpromazine for 14 days	Increased prolactin response to TRH following chlorpromazine treatment

Brambilla et al., 1976 [79]	TRH, 500 mcg i.v.	20 patients with chronic hebephrenic schizophrenia, off medication for at least 10 days; 8 healthy controls	Enhanced prolactin responses to TRH in patients, despite normal basal prolactin levels

Naber et al., 1980 [80]	TRH, 0.2 mg and luteinizing-hormone releasing hormone (LHRH), 0.025 mg	22 patients with chronic schizophrenia (10 male, 12 female) receiving antipsychotics for 6–21 years	Attenuated prolactin response to TRH with long-term treatment; no relation between TRH response and psychopathology

Mokrani et al., 2000 [13]	Clonidine, 0.35–0.375 mg depending on body weight	134 drug-free in-patients—31 schizophrenia, 16 schizoaffective, 87 major depression—and 22 controls	Blunted prolactin response to clonidine in patients with paranoid schizophrenia, compared to controls and patients with disorganized schizophrenia

Sharma et al., 1999 [25]	Fenfluramine, 60 mg orally	35 drug-free patients—28 schizophrenia, 7 schizoaffective	Significant positive correlation between prolactin response to fenfluramine and negative symptoms, as measured by the BPRS

Monteleone et al., 1999 [30]	Fenfluramine, 30 mg orally	16 drug-free patients with schizophrenia; 16 matched controls	Greater prolactin response to fenfluramine in patients with resistant schizophrenia as per Kane's criteria

Abel et al., 1996 [26]	d-Fenfluramine	13 drug-naïve patients with schizophrenia; 13 matched controls	Greater prolactin response in patients; positive correlation of prolactin response with affective symptoms—anxiety, guilt, and depression—measured on the BPRS

Lerer et al., 1988 [14]	Fenfluramine, 60 mg orally	10 drug-free patients with “chronic schizophrenia”; 10 matched controls	Blunted prolactin response to fenfluramine in patients

Krystal et al., 1993 [81]	m-Chlorophenylpiperazine (m-CPP), 0.1 mg/kg i.v.	12 drug-free patients with schizophrenia; 15 controls	Lower baseline prolactin in patients; no difference in m-CPP response; m-CPP triggered positive symptoms in patients

Iqbal et al., 1991 [15]	m-CPP, 0.25 mg/kg orally	7 male patients with schizophrenia; 8 male controls	Blunted prolactin response to m-CPP in patients; m-CPP worsened positive symptoms in this group

Markianos et al., 2002 [82]	Clomipramine 25 mg i.v.	25 male patients with schizophrenia, pre- and posttreatment with clozapine or olanzapine	Significant increases in prolactin following clomipramine; this response was blocked by both drugs

Cowen et al., 1985 [83]	L-tryptophan, 7.5 g i.v.	18 patients with schizophrenia on treatment and healthy controls	Increased prolactin response to L-tryptophan in patients

Nerozzi et al., 1990 [84]	Growth hormone-releasing hormone (GHRH), 1 mcg/kg	18 drug-free male patients with schizophrenia; 18 matched controls	Transient increase in prolactin following GHRH; no difference between the two groups

Cantalamessa et al., 1985 [85]	Gonadotrophin-releasing hormone (GnRH)	11 male patients with “acute schizophrenia”	Increased prolactin in 2 of 11 patients following GnRH

Keks et al., 1990, 1992 [16, 17]	Haloperidol, 0.5 mg i.v.	44 drug-free male patients with “acute schizophrenia”; 28 healthy controls	Blunted prolactin response to haloperidol in patients; basal prolactin positively correlated with the BPRS depression score. Maximal blunting seen in patients diagnosed with schizophrenia as per Kraepelin's criteria

Copolov et al., 1990 [18]	Low-dose haloperidol, i.v.	46 male in-patients with psychosis (27 schizophrenia, 7 affective, 12 other psychoses); 28 male controls	Lower prolactin response in patients with schizophrenia than in controls, even after correcting for age

Kulkarni et al., 1990 [29]	Haloperidol, 0.5 mg i.v.	24 drug-free male patients with psychosis	Significant inverse correlation between prolactin response and the severity of delusions

Keks et al., 1987 [19]	Haloperidol, 0.5 mg i.v.	14 drug-free male patients with schizophrenia; 14 matched controls	Blunted and slow prolactin response to haloperidol in patients

Nerozzi et al., 1990 [20]	Domperidone, 20 mg	16 drug-free male patients with schizophrenia or schizophreniform disorder; 16 male controls	Blunted prolactin response to domperidone in all patients, more prominently in the schizophreniform group. Basal prolactin was comparable to controls

Nerozzi et al., 1992 [86]	Domperidone, 20 mg	17 elderly, drug-free male patients with schizophrenia; 8 age-matched male controls	Increased prolactin response to domperidone in patients; comparable baseline prolactin

Monteleone et al., 1985 [21]^#^	Sodium valproate, 800 mg	18 women with “chronic schizophrenia”; 20 healthy women	Valproate suppressed prolactin in normal women, but failed to do so in those with schizophrenia

Scheinin et al., 1985 [22]^#^	Apomorphine, 0.005 mg/kg s.c. and 0.015 mg/kg i.v.	11 patients with schizophrenia on medication; 8 controls	Apomorphine significantly decreased prolactin in controls but not in patients

Whalley et al., 1984 [87]^#^	Apomorphine, 0.75 mg	19 drug-free patients with psychosis; 9 healthy controls	Increased suppression of prolactin by apomorphine in patients with schizophrenia compared to controls

Ferrier et al., 1984 [31]	Apomorphine, 0.75 mg s.c.	Unmedicated patients with acute and chronic schizophrenia; healthy controls	No difference in the response to apomorphine across groups; however, basal prolactin was negatively correlated with the severity of positive symptoms

Rotrosen et al., 1978 [23]	Apomorphine and L-dopa	Unmedicated patients with chronic schizophrenia; healthy controls	Slightly blunted suppression of prolactin in patients compared to controls with apomorphine; greater suppression in patients than controls with L-dopa

Kolakowska et al., 1981 [88]	Apomorphine 0.05–0.1 mg/kg	17 patients with schizophrenia on treatment	No relationship between antipsychotic dose and apomorphine-induced prolactin suppression

Tsuchiya 1984 [24]	Experimental stress	Patients with schizophrenia, hypomania, depression, and alcohol dependence; healthy controls	Stress-induced prolactin release significantly greater in patients with schizophrenia than in controls

^#^Study uses an agent that inhibits prolactin release.

**Table 4:** Studies examining the relationship between measures of prolactin and antipsychotic response in schizophrenia.

Study and authors	Patient sample and drug used	Results
Gruen et al., 1978 [89]	15 patients with schizophrenia; high-dose butaperazine or loxapine for 6 weeks	Transient prolactin elevation during the untreated admission period; consistent elevation in all patients following treatment but no relationship between levels and response

Markianos et al., 1991 [90]	12 patients with schizophrenia; haloperidol 30–60 mg for one month	Increases in baseline prolactin were significantly correlated with reduction in the BPRS total score

Zhang et al., 2002 [42]	30 male patients with “chronic schizophrenia”; risperidone 6 mg/day for 12 weeks	A significant association was found between increases in prolactin level and the positive subscore of the Positive and Negative Symptom Scale (PANSS)

Zhang et al., 2005 [91]	78 in-patients with schizophrenia; randomized to risperidone 6 mg/day or haloperidol 20 mg/day for 12 weeks	Change in prolactin levels was significantly related to improvement in the PANSS positive score in patients on risperidone but not haloperidol

Otani et al., 1994 [92]	24 patients with schizophrenia (12 male, 12 female); zotepine 100 mg/day for 1 week, then 200 mg/day for 3 weeks	Significant relationship between increases in prolactin and positive symptom improvement as measured by the BPRS but only in male patients

Kitamura et al., 1988 [93]	100 patients (96 with schizophrenia) receiving fluphenazine decanoate for at least 12 weeks	Higher prolactin to plasma fluphenazine ratio was associated with a better outcome (out-patient versus in-patient status)

Larsson et al., 1984 [94]	4 patients with schizophrenia; thioridazine with or without alpha-methyl tyrosine, 2 g/day	Positive correlation between the antipsychotic response to thioridazine and changes in prolactin

Chou et al., 1998 [95]	23 patients with acute exacerbations of schizophrenia or schizoaffective disorder; haloperidol titrated to achieve “low to moderate” plasma levels for 3 weeks	Increases in prolactin were associated with fewer symptoms at the end of treatment

Van Putten et al., 1991 [33]	73 drug-free male in-patients with schizophrenia; haloperidol 5–20 mg/day for 4 weeks	Posttreatment prolactin levels were significantly related to treatment outcome, but this reached a plateau at levels of 30 ng/mL (achieved with a dose of around 10 mg/day)

Mohr et al., 1998 [96]	23 in-patients (11 male, 12 female) with schizophrenia; haloperidol for 4 weeks	An exaggerated basal prolactin response to TRH predicted a worse response to haloperidol across several domains (positive, negative, anxiety-depression, and total BPRS scores)

Jones et al., 1998 [41]	20 patients with schizophrenia; clozapine	Blunting of the prolactin response to fenfluramine after clozapine treatment was significantly correlated with reductions in total positive symptoms, delusions, and hallucinations as measured by the Scale for the Assessment of Positive Symptoms (SAPS)

Lieberman et al., 1994 [97]	Patients with schizophrenia; clozapine	Blunting of the prolactin response to apomorphine after clozapine treatment was associated with a better response

Meltzer and Busch, 1983 [98]	Drug-free patients with schizophrenia; chlorpromazine 100 mg bid for 1 week and 200 mg bid for 1 week	At doses of 200 mg of chlorpromazine, prolactin elevation was negatively correlated with the severity of hallucinations

Awad et al., 1990 [43]	Patients with schizophrenia; remoxipride (high- and low-dose) and haloperidol	Higher basal prolactin predicted responses to both drugs but only in male patients

Wang et al., 2007 [34]	118 patients with schizophrenia (78 female, 40 male); risperidone 2–8 mg for 8 weeks	No relationship between changes in plasma prolactin and the response to risperidone

Volavka et al., 2004 [35]	157 patients with treatment-resistant schizophrenia (133 male, 24 female); randomized to clozapine, olanzapine, risperidone, or haloperidol for 14 weeks	No relationship between prolactin levels and clinical improvement

Rimon et al., 1985 [37]	28 patients with “acute symptoms” of schizophrenia; fluphenazine for 8 weeks	No correlation between changes in either plasma or CSF prolactin and response to fluphenazine

Smith et al., 1984 [99]	Patients with schizophrenia; haloperidol	No relationship between changes in prolactin level and response to haloperidol

Meltzer et al., 1983 [36]	21 drug-free in-patients with schizophrenia (10 female, 11 male); chlorpromazine 200 mg for 1 week, then 400 mg for 1 week	No relationship between prolactin levels and clinical outcome

Meco et al., 1983 [38]	23 out-patients (11 male, 12 female) with schizophreniform disorder; haloperidol decanoate 50–250 mg once in 4 weeks for 12 months	No relationship between prolactin levels and clinical improvement

Jørgensen et al., 1982 [39]	9 patients with chronic schizophrenia; flupenthixol decanoate	No correlation between prolactin levels and changes in clinical ratings

Kolakowska et al., 1979 [40]	19 patients with acute psychoses, including schizophrenia; chlorpromazine	Plasma prolactin did not differentiate between patients with a good or poor treatment outcome

**Table 5:** Studies examining the relationship between prolactin and tardive dyskinesia.

Study and authors	Patient sample	Results
Glazer et al., 1981 [100]	19 men with TD; 29 postmenopausal women with TD; 21 men without TD	Prolactin levels were higher in women, but not in men, with severe TD compared to those with mild TD

Csernansky et al., 1986 [75]	33 male patients with schizophrenia on treatment; 8 off treatment; 18 normal male controls	“Prolactin index” (plasma prolactin divided by plasma neuroleptic activity) was negatively correlated with the severity of TD in younger patients

Monteleone et al., 1988 [46]	9 patients with schizophrenia and TD; 7 with schizophrenia alone; 10 healthy controls; challenge with sodium valproate 800 mg	Patients with schizophrenia and TD, but not schizophrenia alone, showed a decrease in prolactin following valproate administration; this decrease was correlated with the severity of TD as measured by the Abnormal Involuntary Movement Scale

Shim et al., 2005 [45]	Patients with schizophrenia with and without TD; challenge with the serotonin agonist and dopamine antagonist buspirone	Prolactin response to buspirone decreased in patients with TD

Asnis et al., 1979 [101]	6 patients with tardive dyskinesia, on and off medication, and following a challenge with haloperidol 0.5 mg i.m.; healthy controls	No difference in prolactin levels between the groups in any of the conditions

Ettigi et al., 1976 [102]	17 patients with “chronic schizophrenia” (4 of whom had oral TD) and 21 normal controls; challenge with apomorphine 0.75 mg s.c.	No difference in prolactin levels between patients and controls, either baseline or after apomorphine challenge

**Table 6:** Studies of prolactin and circadian rhythm in schizophrenia.

Study and authors	Patient sample	Results
Viganò et al., 2001 [103]	13 patients with schizophrenia (5 drug-free)	Abnormal elevation of diurnal prolactin levels found in 10 of 13 patients

Rao et al., 1994 [104]	115 patients with schizophrenia (90 drug-free, 25 on medication); 34 healthy controls	Significant phase advance in prolactin secretion, similar to that seen in depression

Rao et al., 1993 [105]	Patients with schizophrenia (drug-free and on medication); healthy controls	Lower daily mean and amplitude of prolactin secretion in drug-free female patients with schizophrenia compared to controls

Van Cauter et al., 1991 [48]	9 drug-free male patients with schizophrenia; 9 male controls	Threefold enhancement in the sleep-related increase in prolactin in patients compared to controls

Kemali et al., 1985 [106]	23 in-patients with schizophrenia, off drugs for 2 weeks; control in-patients with “neurotic disorders”	No difference in 24-hour prolactin secretion between the two groups

Appelberg et al., 2002 [49]	17 drug-free patients with nonaffective psychosis	Prolactin strongly correlated with REM latency and negatively correlated with REM sleep in patients
